# SNHG8 is identified as a key regulator of epstein-barr virus(EBV)-associated gastric cancer by an integrative analysis of lncRNA and mRNA expression

**DOI:** 10.18632/oncotarget.13167

**Published:** 2016-11-07

**Authors:** Tao Huang, Yan Ji, Dan Hu, Baozheng Chen, Hejun Zhang, Chao Li, Gang Chen, Xingguang Luo, Xiong-wei Zheng, Xiandong Lin

**Affiliations:** ^1^ Department of Pathology, Fujian Provincial Cancer Hospital and Fujian Medical University Cancer Hospital, Fuzhou, Fujian, China; ^2^ Institute of Health Sciences, Shanghai Institutes for Biological Sciences, Chinese Academy of Sciences and Shanghai Jiao Tong University School of Medicine, Shanghai, China; ^3^ Department of Psychiatry, Yale University School of Medicine, New Haven, CT, USA; ^4^ Fujian Provincial Key Laboratory of Translational Cancer Medicine, Fuzhou, Fujian, China

**Keywords:** gastric cancer, epstein–barr virus, long non-coding RNA, biomarker, SNHG8

## Abstract

The Epstein–Barr virus (EBV) is associated with a variety of cancers, including gastric cancer, which has one of the highest mortality rates of all human cancers. Long non-coding RNAs (lncRNAs) have been suggested to have important causal roles in gastric cancer. However, the interaction between lncRNAs and EBV has not yet been studied. To this end, we sequenced 11,311 lncRNAs and 144,826 protein-coding transcripts from four types of tissue: one non-EBV-infected gastric carcinoma (EBVnGC) and its adjacent normal tissue, and one EBV-associated gastric carcinoma (EBVaGC) and its adjacent normal tissue. Five lncRNAs showed EBVaGC-specific expression; of those, one (SNHG8) was validated using real-time PCR in an independent cohort with 88 paired gastric cancer and adjacent tissue samples. To explore the functions of SNHG8, we identified its mRNA targets on the lncRNA–mRNA co-expression network of the Illumina Body Map, which contains the RNA sequencing data of mRNAs and lncRNAs from 16 normal human tissues. SNHG8 lncRNA was found to affect several gastric cancer-specific pathways and target genes of EBV. Our results reveal the intertwined tumorigenesis mechanisms of lncRNA and EBV and identify SNHG8 as a highly possible candidate biomarker and drug target of gastric cancer.

## INTRODUCTION

Gastric cancer is the fourth most common cancer worldwide and ranks second on the cause list of cancer death. [1]. It is a complex and highly heterogeneous disease. One type of gastric cancer is Epstein–Barr virus (EBV)-associated gastric carcinoma (EBVaGC), which constitutes almost a tenth of all gastric carcinomas [2]. EBV is absent in noncancerous mucosa but present in all cancer cells, and has a clonal nature in neoplastic cells; therefore, it is considered to have a causal role in gastric carcinoma [2, 3]. EBVaGC has been well-characterized molecularly and genomically [4]. However, the pathogenic mechanism of EBVaGC remains poorly understood.

Long non-coding RNAs (lncRNAs) are with ≥ 200 nt but without open reading frames (ORFs). Many studies have demonstrated that lncRNAs have diverse biological functions, such as regulating epigenetic modulation, transcription, and translation [5, 6], and that they are dysregulated in various cancers [7–10]. Furthermore, lncRNAs are being increasingly recognized as master regulators of cancer [6, 8, 11].

In gastric cancer, lncRNA dysregulation is associated with larger tumors, greater tumor invasion, more widespread metastasis, and lower survival rates [12, 13]. For example, expression of the lncRNA PANDAR (promoter of CDKN1A antisense DNA damage activated RNA) is greater in cancerous tissue than in adjacent healthy tissue, and ectopic expression of this promoter is associated with various measures of cancer severity [14]. Another study showed that overexpression of the lncRNA H19 promoted proliferation, migration, invasion, and metastasis of gastric cancer [15]. However, there have been no reports investigating lncRNAs in EBVaGC.

Deep sequencing is a high-throughput technique that enables rapid and comprehensive exploration of a large number of lncRNAs and can be used to identify sequence variations and discover novel lncRNAs [16, 17]. Here, we used deep sequencing to examine the lncRNAs and protein-coding transcripts in four samples: EBVaGC and its adjacent normal tissue, and non-EBV-infected gastric cancer tissue (EBVnGC) and its adjacent normal tissue. Five lncRNAs were specifically expressed in EBVaGC. Analysis of lncRNA and mRNA co-expression and virus–host interactions revealed that the lncRNA SNHG8 interacts with EBV proteins and regulates several important target genes that affect downstream cancer pathways.

## RESULTS

### EBVaGC definition

**Figure 1:**
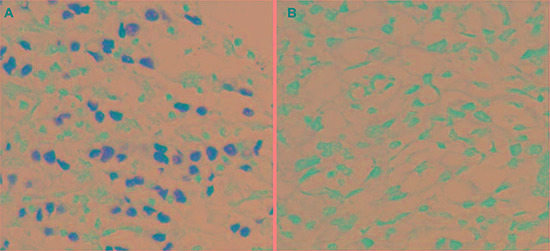
*In situ* hybridization of EBER1 in gastric cancer tissue (**A**) EBVaGC, EBER(+) tissue. (**B**) EBVnGC, EBER(−) tissue. Magnification, ×400. EBER, EBV-encoded small RNA; EBVaGC, EBV-associated gastric carcinoma; EBVnGC, non-EBV-infected gastric cancer

EBVaGC is a lymphoepithelioma-like, diffuse-type carcinoma with dense lymphocytic infiltration. It is identified by the expression of EBV-encoded small ribonucleic acid 1 (EBER1) in cancer cell nuclei, using *in situ* hybridization. Lymphoid stroma surrounds the EBER1-positive nuclei (Figure 1).

### Whole genome lncRNA and mRNA expression profiles

Ultra-high-depth RNA sequencing data sets were generated from two tumor samples (EBVaGC sample with 70.6 million pair-end reads; EBVnGC sample with 65.2 million pair-end reads) and two matched normal samples (EBVaGC adjacent sample with75.5 million pair-end reads; EBVnGC adjacent sample with 53.5 million pair-end reads).

A Trim Galore! Cutadapt wrapper (v1.9.dev6) was used to trim raw sequencing reads, and low quality bases (< Q20) were removed using Trimmomatic v0.32 [18]. FastQC v0.11.3 was used to evaluate the qualities of the raw sequencing data and the trimmed data by analyzing base quality, GC content and sequence length distribution. TopHat2 v2.1.1 [19] was used to align the trimmed reads to the human genome (GRCh37) with reference annotation from Gencode v19. More than 90% of reads were mapped and over 85% of reads were uniquely mapped. rRNA genes were masked, and Cufflinks v.2.2.1 [20] was used to generate transcriptome assemblies. Transcript-level expression was measured as FPKM (fragments per kilobase of exon per million fragments mapped). The average numbers of expressed genes and transcripts (FPKM > 1) were 14,360 and 24,505, respectively.

### EBV-specific lncRNAs

The lncRNAs specifically expressed in EBVaGC tissue were identified using criteria of ≥ 5-fold change between FPKM values of the EBVaGC sample and the other three tissues (EBVnGC sample, EBVaGC adjacent sample, EBVnGC adjacent sample). Five EBVaGC-specific lncRNAs were identified: RNU12, H19, SNHG8, RP11-359D14.3, and MIR143HG (Table 1).

**Table 1:** The FPKM expression levels of EBV-specific lncRNAs

Transcript ID	Transcript Name	EBV-negative tumor sample (EBVnGC)	EBV-negative adjacent sample	EBV-positive tumor sample (EBVaGC)	EBV-positive adjacent sample
ENST00000362512	RNU12	321.19	138.32	1648.37	264.21
ENST00000414790	H19	0.49	1.99	30.63	0.98
ENST00000412788	H19	0.00	0.00	6.73	0.52
ENST00000449007	RP11-359D14.3	0.00	0.00	5.19	0.00
ENST00000384096	SNHG8	0.00	0.00	4.21	0.01
ENST00000522358	MIR143HG	0.24	0.37	3.03	0.00

### Polymerase chain reaction validation of the EBV-specific lncRNA SNHG8

**Figure 2:**
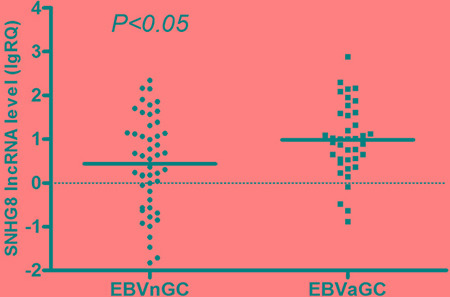
Distribution of SNHG8 lncRNA levels in EBVnGC and EBVaGC Bold lines represent the mean value for each patient cohort; RQ = 2^**−**ΔΔCt^

**Figure 3:**
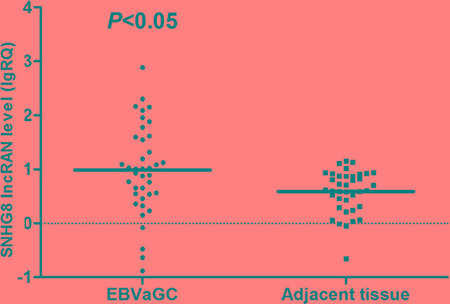
Distribution of SNHG8 lncRNA levels in EBVaGC and adjacent tissue Bold lines represent the mean value for each patient cohort; RQ = 2^−ΔΔCt^

Real-time reverse transcription polymerase chain reaction (RT-PCR) analysis was used to further validate the expression levels of the five EBV-specific lncRNAs in a cohort of 88 patients with gastric cancer, with primers designed in Primer Premier 5.0 software (Supplementary Table S1). The RP11-359D14.3 primer was difficult to design and was removed after unsatisfactory quality control results. There were no pathological differences between the gastric carcinoma samples used for RNA sequencing and those used for RT-PCR validation (Supplementary Table S2). SNHG8 expression was concordant with the lncRNA sequencing assay. The RT-PCR results of non-significant lncRNAs, RNU12, H19 and MIR143HG are shown in Supplementary Figures S1–S3. Notably, SNHG8 expression in EBVaGC was significantly higher than in EBVnGC (Figure 2) and in EBVaGC adjacent tissue (Figure 3).

### Biological functions of SNHG8 target genes

Next, we identified the target mRNAs of SNHG8 by analyzing its coexpression with mRNAs according to the Illumina Body Map dataset. Using the online gene function annotation tool DAVID [21], which includes numerous annotation categories such as Gene Ontology (GO) and KEGG Pathways, we explored the functions of the target genes of SNHG8 (Table 2). These results indicated that SNHG8 targets pathways such as hsa03010 (ribosome), GO:0006412 (translation), GO:0045449 (regulation of transcription), GO:0006350 (transcription), GO:0008380 (RNA splicing), GO:0016071 (mRNA metabolic process), GO:0008134 (transcription factor binding), GO:0003677 (DNA binding), and GO:0030528 (transcription regulator activity). Notably, many of these functions, such as “transcription” and “mRNA metabolic process”, were well-known pathways or processes targeted by EBV [22–24].

**Table 2:** Significantly enriched KEGG and GO terms of SNHG8 target genes using DAVID

Category	Term	FDR(<0.05)
KEGG PATHWAY	hsa03010:Ribosome	3.77E-13
Gene Ontology (GO) Biological Processes (BP)	GO:0006412~translation	1.54E-14
	GO:0006414~translational elongation	1.25E-11
	GO:0006396~RNA processing	2.91E-05
	GO:0045449~regulation of transcription	0.001145
	GO:0006350~transcription	0.002024
	GO:0008380~RNA splicing	0.040761
	GO:0016071~mRNA metabolic process	0.041671
Gene Ontology (GO) Molecular Function (MF)	GO:0003735~structural constituent of ribosome	6.18E-09
	GO:0003723~RNA binding	2.84E-06
	GO:0008134~transcription factor binding	0.002199
	GO:0003677~DNA binding	0.004828
	GO:0030528~transcription regulator activity	0.012988
Gene Ontology (GO) Cellular Component (CC)	GO:0030529~ribonucleoprotein complex	1.55E-13
	GO:0022626~cytosolic ribosome	5.93E-09
	GO:0005840~ribosome	4.48E-08
	GO:0043232~intracellular non-membrane-bounded organelle	4.91E-07
	GO:0043228~non-membrane-bounded organelle	4.91E-07
	GO:0005829~cytosol	2.56E-05
	GO:0033279~ribosomal subunit	9.32E-05
	GO:0031981~nuclear lumen	3.65E-04
	GO:0022625~cytosolic large ribosomal subunit	8.32E-04
	GO:0044445~cytosolic part	0.001145
	GO:0005730~nucleolus	0.003792
	GO:0031974~membrane-enclosed lumen	0.010549
	GO:0070013~intracellular organelle lumen	0.013856
	GO:0043233~organelle lumen	0.017307
	GO:0005681~spliceosome	0.019806

### Relationship between SNHG8 and EBV in EBVaGC

We further investigated the relationship between SNHG8 and EBV by analyzing the co-expression between host human mRNAs and EBV mRNAs. We used an EBV genomics dataset to identify target mRNAs of EBV genes.

Using the hypergeometric statistical test, we evaluated the overlap between EBV target genes and SNHG8 target genes. The enrichment results (Table 3) show that SNHG8 interacts significantly with EBV genes such as *BHLF1*, *LF3*, *BHRF1*, and *BNLF2a*.

The EBV genes *BHLF1* (BamHI H leftward reading frame 1) and *LF3* (leftward reading frame 3) consist of repetitive sequences of 125 and 102 bp, respectively. They are both found in the polyribosomal fraction of cells infected with EBV and expressed transcriptionally in virus-associated tumors [25]. There is evidence that *BHLF1* and *LF3* are associated with the lytic replication cycle, which seems to take place mainly in epithelial cells. This type of replication is essential for the spread of the virus, and its suppression maintains the tumor phenotype [26].

BHRF1 has 38% primary sequence homology with the antiapoptotic protein Bcl-2, and shares three of its four conserved regions (Bcl-2 homology (BH) domains, BH1–BH3) [27]. The functions of BHRF1 are also similar to those of Bcl-2, and it imparts anti-apoptotic protection to EBV-infected cells [28], allowing the development of virus persistence and facilitating oncogenesis.

BNLF2a is another early lytic gene, and encodes a tail-anchored protein situated in the membrane of the endoplasmic reticulum [29]. The BNLF2a protein has two domains: a hydrophilic, cytosolic N-terminal domain and a hydrophobic, membrane-spanning C-terminal domain [30]. Both domains are required for immune escape, which involves the disruption of viral peptide transport into the endoplasmic reticulum and of peptide loading onto human leukocyte antigen class I molecules; this disruption leads to lower levels of endogenous antigen presentation, thus preventing recognition by CD8+ T-cells [30, 31].

### EBV target genes with the same expression pattern as SNHG8

The target genes of EBV that are also targeted by SNHG8 are listed in Table 3. As described above, SNHG8 expression in EBVaGC was significantly higher than in EBVnGC and adjacent tissues. Next, we explored the target genes for EBV and SNHG8, and identified those that showed the same expression pattern: EBVaGC FPKM expression level ≥ 5 and greater expression than in EBVnGC and in EBVaGC adjacent tissue (Table 4).

TRIM28, also known as KAP1 and TIF1b, is a universal co-repressor that mediates transcriptional control by interacting with Krüppel-associated box zinc finger proteins [32, 33]. TRIM28 is an essential partner in several multiple-protein complexes and has a variety of functions including the regulation of pluripotency and proliferation [34, 35]. It participates in epithelial–mesenchymal transition via the regulation of histone acetylation and methylation on E- and N-cadherin promoters in lung cancer cell lines. TRIM28 is involved in cancer progression; it is overexpressed in colorectal and gastric cancer and is an independent prognostic factor for poor overall and relapse-free survival [36].

The highly conserved gene *EIF4A2* is a member of the eukaryotic initiation factor 4A family, and encodes a protein synthesis initiation factor for binding mRNA to the ribosome. EIF4A2 is involved in the progression of breast cancer and melanoma [37] and in the development of non-small-cell lung cancer, and has been suggested as a potential prognostic marker [38].

Nucleosome assembly protein-1 (Nap1) plays a role in cell proliferation and cell cycle progression, as well as nucleosome assembly [39, 40]. Nap1-like 1 (Nap1L1) is highly homologous to Nap1 and shares some functions with it, such as nucleosome assembly, although it also has a more active role in nucleosome disassembly [40]. Nap1L1 is overexpressed in certain tumors such as hepatoblastoma [41] and carcinoid of the small intestine [42]. It epigenetically promotes tumor cell proliferation in pancreatic neuroendocrine neoplasms by inhibiting the tumor suppressor p57Kip2 and the mTOR pathway [43].

*PLD3* encodes a lipase family protein associated with the endoplasmic reticulum, which is widely expressed in the brain, including in the hippocampus and most of the cortex [44, 45]. PDL3 has been implicated in late-onset Alzheimer's disease and might contribute to a range of cellular functions including differentiation, epigenetic modification, neurotransmission, and signal transduction [44, 45].

Ribosomal protein L18a (RPL18A) is a component of the eukaryotic large ribosomal subunit (60S). RPL18A interacts with the hepatitis C virus internal ribosome entry site (IRES) and might be involved in IRES-mediated translation and viral replication [46, 47].

The channel kinase TRPM7 transduces physical and chemical stress. It has intrinsic kinase activity and is involved in cell growth, proliferation, migration, differentiation, and survival [48, 49]. Aberrant TRPM7 expression is associated with a number of cancers [49–51] including breast carcinoma and head/neck cancer [52–54]. Furthermore, TRPM7 might regulate exocrine pancreatic development, and aberrant TRPM7-mediated signaling contributes to the development of pancreatic cancer [54].

**Table 3:** EBV proteins whose target genes significantly overlapped with SNHG8 targets

EBV protein	FDR(<0.05)	Number of EBV target genes	Number of EBV target genes that were also targeted by SNHG8	EBV target genes that were also targeted by SNHG8
LF3	6.93E-05	300	28	AHDC1, AMBRA1, BAHD1, C19orf26, CENPB, CIC, EEF2, EIF4A2, ELK1, GLTPD1, HNRNPA0, IRF2BP1, KHSRP, KLHL26, MEF2D, MGRN1, MLLT1, NCOR2, NFIC, PLD3, PLIN3, PTPN23, SAMD4B, SART1, SF1, SURF6, ZBTB4, ZBTB7A
BHLF1	0.000252	568	40	AHDC1, AMBRA1, BAD, BAHD1, BTBD2, BTF3, C19orf26, CD58, CENPB, CIC, CLIP2, EEF2, EIF4A2, ELK1, GLTPD1, GTF2F1, GTPBP1, HDGFRP2, HNRNPA0, IRF2BP1, KHSRP, KLHL26, LARP7, MEF2D, MLLT1, MLLT6, MTERFD3, N6AMT1, NFIC, NUDT16L1, PLD3, PLIN3, SAMD1, SAMD4B, SART1, SURF6, TAF7, TRIM28, ZBTB4, ZNF324B
BHRF1	0.008401	793	45	AHDC1, BTBD2, BTF3L4, CD58, CENPB, CIC, COMMD10, CPSF1, EEF2, EIF3G, EIF4A2, ERCC8, GCNT2, GEN1, GLTPD1, GTF2F1, GTF2H2, GTPBP1, HDGFRP2, IRF2BP1, KHSRP, KLHL26, MEF2D, MGRN1, MLLT1, MTERFD3, NAP1L1, NCOR2, NFIC, PLD3, PTPN23, RBM10, RNF14, SAMD1, SAMD4B, SART1, TAF7, TMEM168, TRIM28, TRPM7, ZBTB4, ZBTB7A, ZNF337, ZNF345, ZNF720
BNLF2a	0.039096	40	6	BRD4, DLGAP4, NFKBIL1, RPL18A, TRIP10, WBP2

**Table 4:** The FPKM expression levels of EBV target genes with the same expression pattern as SNHG8

Transcript ID	Transcript Name	EBV-negative tumor sample(EBVnGC)	EBV-negative adjacent sample	EBV-positive tumor sample (EBVaGC)	EBV-positive adjacent sample
ENST00000600840	TRIM28	3.38	5.18	5.18	3.55
ENST00000323963	EIF4A2	0.67	16.67	21.20	18.62
ENST00000496382	EIF4A2	0.00	0.00	5.61	0.00
ENST00000393263	NAP1L1	1.48	2.70	5.53	3.65
ENST00000547773	NAP1L1	1.81	1.47	5.29	2.06
ENST00000409281	PLD3	5.57	0.00	11.27	0.00
ENST00000222247	RPL18A	74.79	150.44	101.37	81.57
ENST00000313478	TRPM7	3.11	2.14	4.20	3.42

## DISCUSSION

In the present study, we have evaluated the profiles of lncRNAs that are aberrantly expressed in EBVaGC, and confirmed expression levels of the lncRNA SNHG8 by RT-PCR. The putative functions of SNHG8 were explored by examining co-expression of lncRNA and mRNAs.

Mounting evidence suggests that lncRNAs, initially considered transcriptional noise, play pivotal roles in carcinogenesis [11, 55]. In gastric cancer, the dysregulation of several lncRNAs is associated with tumorigenesis, metastasis, and prognosis [12, 56]. For example, the Hox transcript antisense intergenic RNA (HOT-AIR), one of the most widely known lncRNAs, was shown to be associated with TNM stage and lymph node metastasis in patients with gastric cancer. HOT-AIR also promotes invasion and epithelial–mesenchymal transition by directing polycomb repressive complex 2 (PRC2) to silence HOXD9 [57, 58]. Homeobox A transcript at the distal tip (HOTTIP) is markedly overexpressed in gastric cancer tissues and associated with several measures of severity including TNM stage and overall survival. Furthermore, overexpression of HOTTIP was identified as an independent poor prognostic factor for patients with gastric cancer. Together, this indicates that lncRNAs are an excellent prospect as a new type of biomarker [56]. Our study also shows that SNHG8 expression was markedly elevated in EBVaGC tissues compared with normal control samples.

It has long been accepted that small nucleolar RNAs (snoRNAs) guide RNA in post-translational ribosomal RNA modification processes [59, 60]. However, accumulating evidence suggests that these non-coding RNAs might play a much more important role in cell fate determination and oncogenesis than previously thought [61, 62]. In patients with gastric cancer, SNHG5 was significantly downregulated and associated with TNM stage [63]. Furthermore, SNHG20 was upregulated in hepatocellular carcinoma and, in an *in vitro* study, its suppression distinctly inhibited hepatocellular carcinoma cell proliferation, migration, and invasion [64]. SNHG8, located on 4q26, is thought to encode the smaller snoRNAs. Our study showed that SNHG8 expression in EBVaGC tissues is markedly elevated compared with normal control samples.

EBV is a lifelong latent infection present in more than 90% of the human population and has been linked etiologically to a wide range of human malignancies [65]. EBV-encoded proteins, such as EBV nuclear antigen 1 and latent membrane proteins, can alter gene expression, accelerate growth, increase survival, and facilitate invasion of transformed cells [66, 67]. A number of viral non-coding RNAs have also been linked to latent EBV infection; for example, EBV BamHI-A rightward transcripts (BARTs), a family of alternatively spliced mRNAs expressed in EBV latency programs, are closely associated with clinical and pathological measures of tumor progression [68]. BART1 induces metastasis via PTEN-dependent pathway regulation [69]; BART3 promotes cell growth by its action on deleted in cancer 1 (DICE1) [70]; and BART5 inhibits apoptosis by modulating the pro-apoptotic protein p53 upregulated modulator of apoptosis (PUMA) [71]. This suggests that EBV plays a causal role in the development of malignancies, metastasis of tumors, and evasion of the host immune system.

EBVaGC has unique clinicopathologic characteristics, including better prognosis than EBVnGC. Several well-recognized viral genes such as *BHRF1*, *BLLF1*, *BRLF1*, *BZLF1*, *EBNA1*, and *LMP2A* are highly expressed in EBVaGC [72, 73]. Expression of LMP2A is involved in the upregulation of survivin protein and causes genome-wide aberrant methylation in host cells [74]. Patients with EBVaGC show typical genetic and epigenetic alterations, and approximately 205 host cell genes are usually mutated including *AKT2*, *CCNA1*, *MAP3K4*, and *TGFBR1* [74].

**Figure 4:**
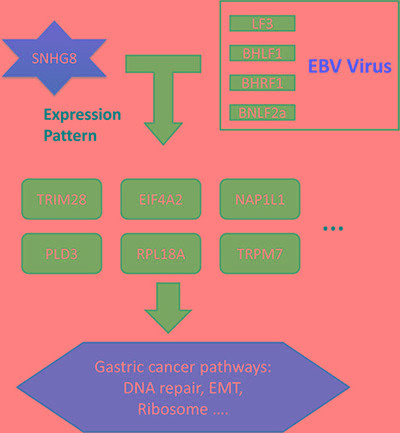
SNHG8, EBV, and their targets in EBV-associated gastric cancer Co-expression and enrichment analysis showed that SNHG8 interacted with the EBV proteins LF3, BHLF1, BHRF1, and BNLF2a. These proteins in turn regulated the expression of TRIM28, EIF4A2, NAP1L1, PLD3, RPL18A, and TRPM7, which play important roles in gastric cancer pathways, contributing to processes such as DNA repair, epithelial–mesenchymal transition, and ribosomal function

In our study, the lncRNA SNHG8 was expressed in an EBV-specific manner. SNHG8 expression in EBVaGC was higher than in EBVnGC and in EBVaGC adjacent tissue. Based on our analysis of SNHG8 and EBV targets, we propose a theory of how SNHG8 triggers gastric cancer (Figure 4). SNHG8 interacts with the EBV proteins LF3, BHLF1, BHRF1, and BNLF2a and regulates the expression of TRIM28, EIF4A2, NAP1L1, PLD3, RPL18A, and TRPM7. Functional analysis of TRIM28, EIF4A2, NAP1L1, PLD3, RPL18A, and TRPM7 suggested that they play direct roles in gastric cancer. This reveals the regulatory roles of lncRNAs and viruses in gastric carcinoma, and contributes to a more comprehensive understanding of tumorigenesis.

## MATERIALS AND METHODS

### Patient and tissue samples

### RNA deep sequencing samples

Two gastric cancer tissues (one EBVnGC and one EBVaGC) and their pair-matched adjacent gastric tissues were obtained from two patients at Fujian Provincial Cancer Hospital. The patients were male with poorly differentiated stage IIIB adenocarcinoma and lymph node metastasis.

### Validated samples

Eighty-eight patients, including 39 with EBVaGC, were included in this study. They took gastric carcinoma resection from July 2012 to April 2015. The patients (69 males and 19 females) had a median age of 58.2 years (ranging from 22.0 to 79.0 years) and had a median tumor size of 5.5 cm (1.0–15.0 cm). None of them received chemotherapy before surgery. Fresh stomach tumor tissues and their adjacent non-tumorous tissues were obtained immediately after tumor resection. One portion of the tissues was immediately snap-frozen in liquid nitrogen and then stored at −80°C; and the other portion was fixed in 10% buffered formalin and then embedded in paraffin. Lauren's criteria [75] was used to determine the histologic subtypes of the tumors. EBVaGC was identified by *in situ* hybridization for EBER1 (Dako, Denmark) (Figure 1) [76].

The study was approved by the Research Ethics Committee of the Fujian Provincial Cancer Hospital, China. Informed consent from all patients was obtained before participation.

### Sequencing and assembly

Total RNA was isolated using Trizol reagent (Invitrogen, Carlsbad, CA, USA). RNA was examined by gel electrophoresis and only high quality RNA was used for subsequent analysis. RNA-Seq libraries were prepared using an Illumina HiSeq 3000 sequencing system with a 50 bp single-end protocol (Illumina, Inc., San Diego, CA, USA) [77]. In total, there were 52 and 59 million 2 × 150 paired-end reads of the paired GC/control mucosa RNA samples [77, 78].

The raw sequencing reads were analyzed with Trim Galore! Cutadapt wrapper v1.9.dev6 (http://www.bioinformatics.babraham.ac.uk/projects/trim_galore/) and Trimmomatic v0.32 [18]: the adapters were trimmed with the Trim Galore! Cutadapt wrapper using the –paired option, and low quality bases (< Q20) were removed with Trimmomatic. FastQC v0.11.3 (http://www.bioinformatics.babraham.ac.uk/projects/fastqc/) was used to manually evaluate the qualities of the raw and trimmed sequencing data by checking per base quality, per base GC content and sequence length distribution. TopHat2 v2.1.1 [19] was used to align the trimmed reads to the human genome (GRCh37) with reference annotation from Gencode v19. More than 90% reads were mapped while over 85% reads were uniquely mapped. rRNA genes were masked, and transcriptome assemblies were generated using Cufflinks (version 2.2.1) [20].

### Criteria for defining EBV-specific lncRNAs

EBV-specific lncRNAs were defined by fold changes (≥ 5) between FPKM values of the EBV-positive tumor sample and the other three samples.

### Real-time RT-PCR assay

Quantitative RT-PCR was used to validate the sequencing results. Total RNA from 88 paired gastric cancer and adjacent tissues was treated with DNaseI (Sigma, St Louis, MO, USA) to eliminate any genomic DNA contamination. Reverse transcription for lncRNAs was performed using M-MLV Reverse Transcriptase (Takara, Japan). The cDNA template was amplified by real-time RT-PCR using the SYBR Green Master Mix (Roche, USA). Primers were designed using Primer Premier 5.0 software. Real-time RT-PCR reactions were performed in triplicate on the ABI7500 system (Applied Biosystems, CA, USA).

Using the comparative Ct method 2^−ΔΔCt^ [79, 80] and gastric carcinoma sample No. 9 as a calibrator, the relative expression levels in all gastric carcinoma samples and adjacent non-tumorous tissues were quantified. Expression levels of lncRNA were normalized to β-actin mRNA expression.

### Identification of mRNA targets of lncRNAs

To identify the mRNA targets of lncRNAs, we analyzed the RNA sequencing dataset of the Illumina Body Map [81], which included 16 normal human tissues. Expression levels of 14,886 lncRNAs from the LNCipedia database [82] and 21,721 protein-coding genes from UCSC hg19 [83] were calculated using TopHat [84] and Cufflinks [85] with default parameters. If the expression level of a protein-coding gene was correlated with that of a lncRNA with an absolute Pearson correlation coefficient > 0.5, they were deemed a co-expression pair. The co-expressed mRNAs were considered to constitute the microenvironment around the lncRNA and were used to annotate the functions of the lncRNA.

### Identification of human target genes of EBV proteins

The target genes of EBV proteins were obtained from EBV Genomics (https://ebv.wistar.upenn.edu)[86]. We downloaded the human gene expression levels and EBV expression levels in 201 samples, and then calculated the Pearson correlation coefficient between the human and EBV genes. Human genes with an absolute Pearson correlation coefficient > 0.5 were considered as the target genes of an EBV gene.

### Enrichment between lncRNA and EBV

Enrichment between lncRNA and EBV genes can be represented by the hypergeometric test *P value* [87–89] of the target gene of lncRNA, L(i), and the target gene of EBV, V(j), which can be computed by:

(1) $$\text{p}\left(\text{i,j}\right)=\text{p}\left(\text{L}\left(\text{i}\right),\text{V}\left(\text{j}\right)\right)=\sum_{k=m}^{n}\frac{\left(\begin{array}{c} M\\ m \end{array}\right)\left(\begin{array}{c} N-M\\ n-m \end{array}\right)}{\left(\begin{array}{c} N\\ n \end{array}\right)}$$

where *N* represents the total number of human genes, *M* and *n* represent the number of target genes of EBV gene j and the number of target genes of lncRNA i, respectively, and *m* represents the number of lncRNA target genes that also target genes of EBV gene j. The smaller the *P value* for a lncRNA and an EBV gene, the stronger the suggested association between them. The hypergeometric test *P* value was adjusted to the false discovery rate using the Benjamini method [90]. A false discovery rate of < 0.05 was considered statistically significant.

### Statistical analysis

Statistical analyses were conducted using IBM SPSS Statistics 19. The two-tailed Student's *t* test was used to identify differentially expressed lncRNAs between EBVaGC and non-EBVaGC. *P* < 0.05 was considered statistically significant.

## CONCLUSIONS

Gastric cancer is an important malignancy with high morbidity and mortality rates and many risk factors. EBV is known to occur often in gastric cancer samples, but certain lncRNAs are also emerging as risk factors for cancer, although their precise roles in the disease remain unclear. To identify the key lncRNAs and investigate their functions and interactions with EBV, we sequenced one EBVnGC tissue and its adjacent normal tissue, and one EBVaGC and its adjacent EBV-associated tissue. The lncRNA SNHG8 was expressed in an EBV-specific manner. Co-expression network analysis revealed significant interactions of SNHG8 and EBV LF3, BHLF1, BHRF1, and BNLF2a. Together, these factors regulate several functional genes in gastric cancer, such as *TRIM28*, *EIF4A2*, *NAP1L1*, *PLD3*, *RPL18A*, and *TRPM7*. This regulatory pathway model of lncRNA, virus, and target genes provides novel insights into gastric tumorigenesis and suggests potential drug targets for intervention.

## SUPPLEMENTARY MATERIALS


