# Supplementary material for: SNHG8 is identified as a key regulator of epstein-barr virus(EBV)-associated gastric cancer by an integrative analysis of lncRNA and mRNA expression
Source: Oncotarget. 2016 Nov 7;7(49):80990–1002. doi: 10.18632/oncotarget.13167 (PMC5348371; doi:10.18632/oncotarget.13167)
Supplement: Supplementary file 1 [file oncotarget-07-80990-s001.pdf]

SNHG8 is identified as a key regulator of epstein–barr virus(EBV)--associated gastric cancer by an integrative analysis of lncRNA and mRNA expression

Supplementary Materials

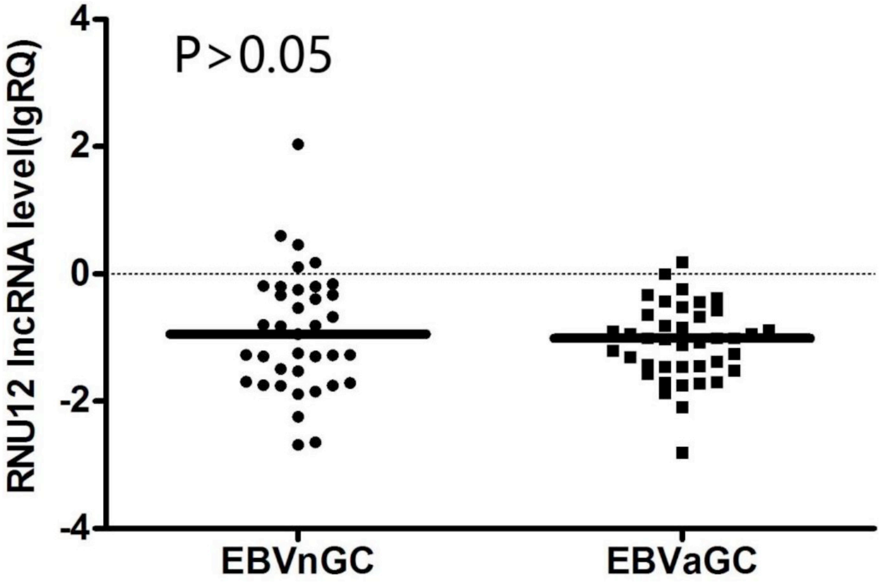

Supplementary Figure S1: Distribution of RNU12 lncRNA levels in EBVnGC and EBVaGC. Bold lines represent the mean value for each patient cohort; RQ=$2^{-\Delta\Delta Ct}$.

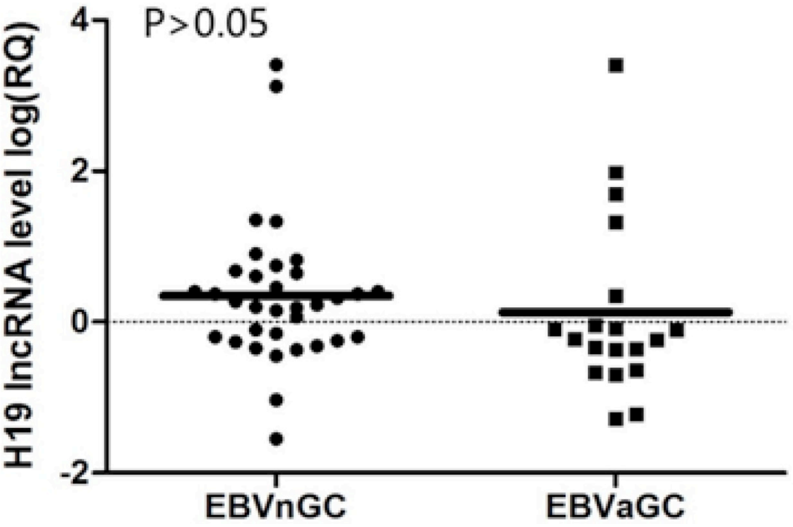

Supplementary Figure S2: Distribution of H19 lncRNA levels in EBVnGC and EBVaGC. Bold lines represent the mean value for each patient cohort; RQ=$2^{-\Delta\Delta Ct}$.

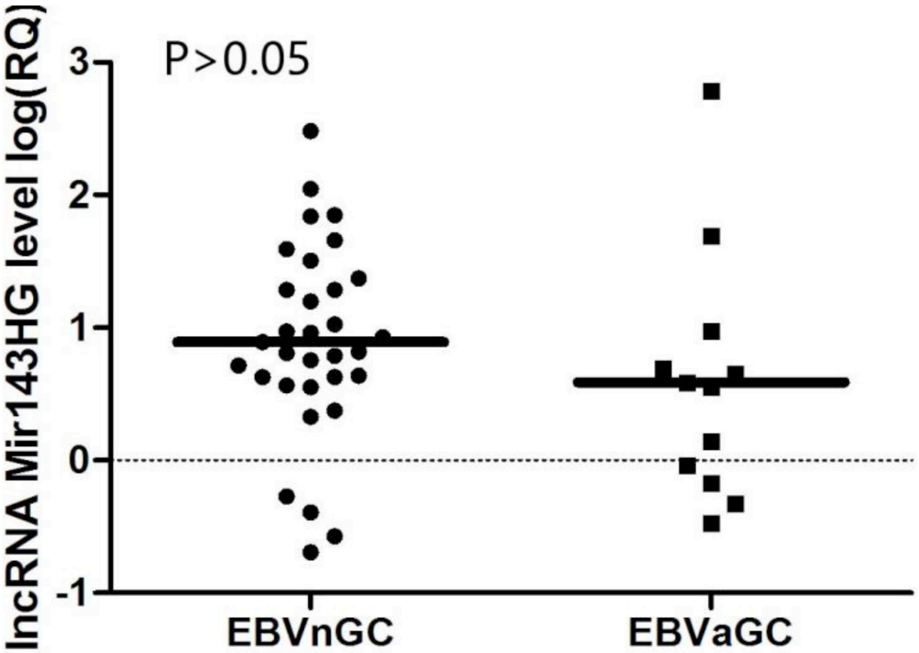

Supplementary Figure S3: Distribution of MIR143HG lncRNA levels in EBVnGC and EBVaGC. Bold lines represent the mean value for each patient cohort; RQ=$2^{-\Delta\Delta Ct}$.

Supplementary Table S1: Primer and product of LncRNAs

GENE	Forward primer	Reverse primer	Product	AnnealT
SNHG8	AAGTTTACAAGCATGCGCGG	TCAAACTGACGGTTCTCGGG	223 bp	60°C
RNU12	ATAACGATTCGGGGTGACGC	ACCTTGAGGGCGACCTTTAC	80 bp	55°C
H19	ATCGGTGCCTCAGCGTTCGG	CTGTCCTCGCCGTCACACCG	145 bp	60°C
MIR143HG	AGAAGACAGAGGTCCCCCAC	CAGTTTTACCTGGCGGCTCT	135 bp	60°C
β-actin	GCGTGACATTAAGGAGAAGC	CCACGTCACACTTCATGATGG	236 bp	60°C

Supplementary Table S2: Pathological features of gastric carcinoma for RNA sequencing and validated samples

Pathological parameters	RNA sequencing samples	Validated samples
**Sex**		
male	2	69
female		19
**Age**		
< 60		48
≥ 60	2	40
**Location**		
EGJ[a]		21
Non-EGJ	2	67
**Depth of invasion**		
< T2		12
≥ T2	2	76
**Lauren's type**		
Intestinal-type		28
diffuse-type	2	60
**Tumor Size**		
< 5 cm		42
≥ 5 cm	2	46
TNM stage		
I+II		23
III+IV	2	65
**LN metastasis**		
absent		24
present	2	64
**EBV infection**		
absent	1	49
present	1	39
